# *Enteroccocus pallens* as a potential novel human pathogen: three cases of spontaneous bacterial peritonitis

**DOI:** 10.1099/jmmcr.0.005024

**Published:** 2016-02-23

**Authors:** Simon Lévesque, Yves Longtin, Marc-Christian Domingo, Cynthia Massé, Harold Bernatchez, Christiane Gaudreau, Cécile Tremblay

**Affiliations:** ^1^​ Laboratoire de santé publique du Québec/Institut national de santé publique du Québec, Sainte-Anne-de-Bellevue, Québec, Canada; ^2^​ SMBD – Jewish General Hospital, Québec, Canada; ^3^​ Centre hospitalier régional Rimouski-Neigette, Québec, Canada; ^4^​ Centre Hospitalier de l'Université de Montréal, Québec, Canada; ^5^​ Centre de recherche de l'Université de Montréal, Québec, Canada

**Keywords:** *Enteroccocus pallens*, liver cirrhosis, peritonitis, Quebec

## Abstract

**Introduction:**

*Enterococcus pallens* is one of the four yellow-pigmented members of the genus *Enterococcus*. To date, a single report of *E. pallens* isolated from a human sample has been published.

**Case presentation:**

We report three cases of *E. pallens* spontaneous bacterial peritonitis in patients with liver cirrhosis that all occurred in Quebec, Canada. Ascitic fluid analysis revealed the presence of *E. pallens* in culture. Identification was made by classical biochemical testing and MALDI-TOF MS, as well as 16S rRNA and elongation factor (*tuf*) gene sequencing. Two of the three patients recovered after antimicrobial treatment.

**Conclusion:**

This report identifies *E. pallens* as a novel human pathogen that appears to possess particular but as-yet unidentified virulence factors that favour the development of peritoneal fluid infections, as previously reported for other *Enterococcus* species. Clinical microbiologist should be aware of this micro-organism which can be identified by phenotypic and molecular methods.

## Introduction

*Enterococcus pallens* is one of the four yellow-pigmented *Enterococcus*. To date, a single report of *E. pallens* isolated from a human sample has been published (Tyrrell *et al.*, 2002). The type strain (ATCC BAA-351) was obtained from a peritoneal dialysate of a patient from Quebec, Canada, who developed peritonitis secondary to intestinal perforation. Here, we report the first case series of human infections caused by this pathogen.

## Case Reports

Patient 1 was a 72-year-old woman known for cirrhosis secondary to non-alcoholic steatohepatitis (NASH) who was admitted in March 2004 to a hospital in Montreal, Canada, because of a 4 month history of increasing abdominal girth, progressive dyspnoea and exacerbation of leg oedema. Her medical history also included coronary artery disease, hypertension, type 2 diabetes mellitus and nephrolithiasis. On examination, the patient was icteric. Her vital signs were normal. She was alert and oriented. The abdomen was markedly distended but non-tender. Laboratory tests revealed a white cell count of 6.2 × 10^9^ cells l^− 1^. Ultrasonography of the abdomen revealed an echogenic liver, a patent portal vein and moderate ascites. A diagnosis of hydrops secondary to NASH-related liver failure was made, and a 1.5 l day^− 1^ fluid restriction and a diuretic therapy were initiated. On day 14 of hospitalization, the patient complained of mild abdominal pain. Abdominal paracentesis was performed to improve her symptoms; 3 l of straw-coloured fluid were drained. Ascitic fluid analysis revealed a red blood cell count of 103 × 10^6^ cells l^− 1^, a leukocyte count of 62 × 10^6^ cells l^− 1^ and an absence of malignant cells. A differential leukocyte count was not performed. No organism was seen on Gram staining of the fluid, but *E. pallens* was isolated on culture. The patient developed an episode of fever (rectal temperature, 39.1 °C) 1 h after the procedure for which antibiotic therapy consisting of intravenous ticarcillin and clavulanic acid was initiated. Blood cultures were negative. On day 16 of hospitalization, the patient complained of shortness of breath and became hypotensive (systolic pressure 95 mmHg). Abdominal paracentesis was repeated; 2.6 l of cloudy yellowish fluid was removed. The ascitic fluid leukocyte count was 600 × 10^6^ cells l^− 1^ with >70 % neutrophils. Culture of peritoneal fluid once again yielded *E. pallens*. The patient fully recovered after a 7 day course of intravenous ticarcillin and clavulanic acid.

Patient 2 was a 62-year-old male known for liver cirrhosis and refractory ascites requiring drainage every other week. He was admitted in May 2013 to a hospital in Rimouski, Canada, due to abdominal pain, increasing abdominal girth and fever. A presumptive diagnosis of spontaneous bacterial peritonitis (SBP) was made and antibiotic therapy consisting of intravenous ceftriaxone was initiated. His medical history also included chronic partial thrombosis of the portal vein, thrombocytopenia, coagulopathy, chronic kidney disease and dilated cardiomyopathy. On the second day of hospitalization, an abdominal paracentesis was performed. Ascitic fluid analysis revealed a white blood cell count of 1611 × 10^6^ cells l^− 1^ with 91 % neutrophils. Direct examination of the ascitic fluid showed no micro-organisms, but *E. pallens* was isolated on culture. Antimicrobial treatment was switched to imipenem and the patient fully recovered.

Patient 3 was a 62-year-old man with alcoholic liver cirrhosis and portal hypertension who was admitted in July 2013 to a hospital in Montreal, Canada, because of hepatic encephalopathy. He was known for refractory ascites requiring large-volume paracentesis twice monthly. His medical history also included a chronic excoriated umbilical hernia, previous hepatic encephalopathy and lower leg lymphoedema. Ascitic fluid analysis revealed a white blood cell count of 447 × 10^6^ cells l^− 1^ with 62 % neutrophils. A diagnosis of SBP was made and antibiotic therapy consisting of intravenous piperacillin/tazobactam was initiated. *E. pallens* was isolated from the ascitic fluid culture, which prompted the addition of intravenous vancomycin on days 4 and 5 of hospitalization. Blood and urine cultures were negative. Chest radiography showed no consolidation or infiltrate. Repeat abdominal paracentesis was performed on day 3 of hospitalization which showed < 250 × 10^6^ neutrophils l^–1^ and a negative ascitic fluid culture. A third abdominal paracentesis was performed on day 5, which this time showed a very high neutrophil count (2725 × 10^6^ cells l^− 1^) and yielded *Escherichia coli*. The patient died on day 8 due to global deterioration.

## Investigations

**Fig. 1:**
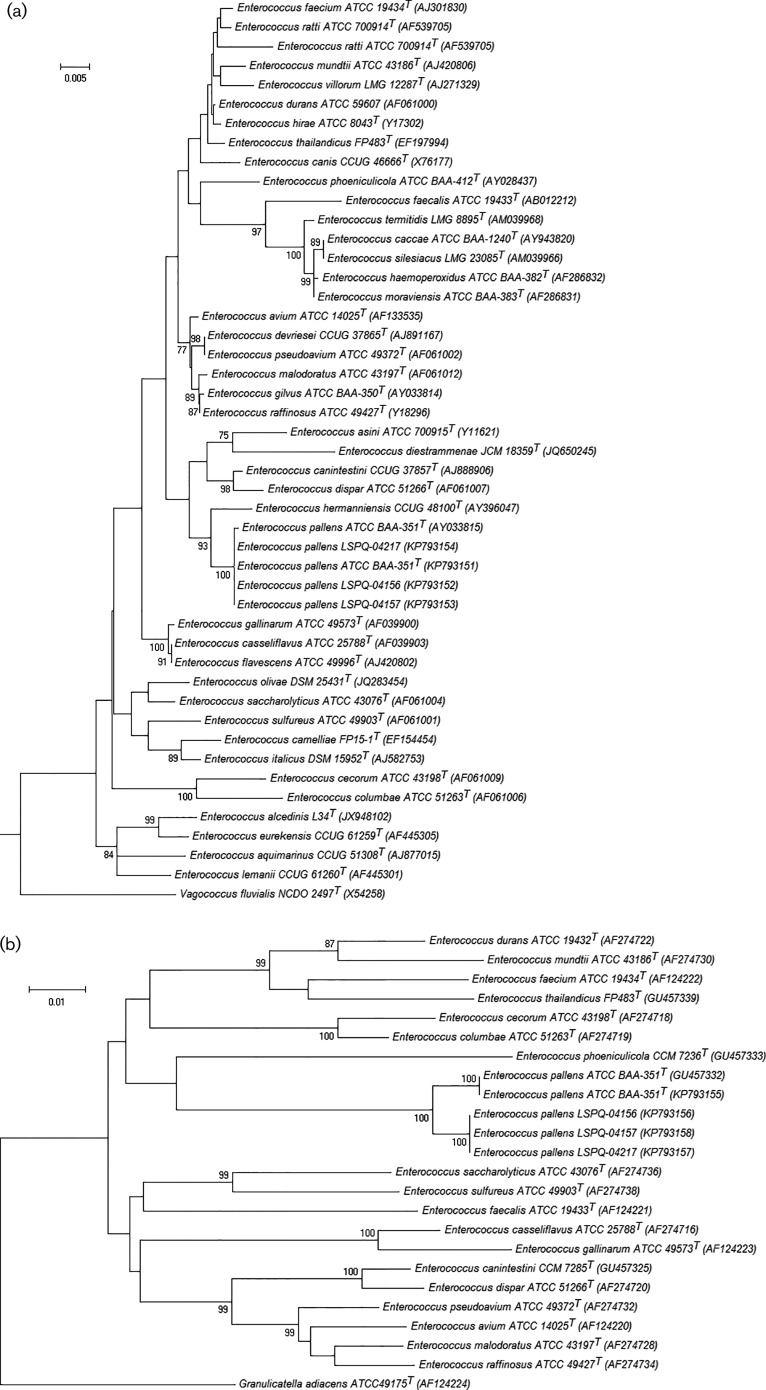
Neighbour-joining phylogenetic tree showing the relationships of the three patient's isolates to related *Enterococcus* species. Bootstrap values were calculated from 1000 replications. Names and GenBank accession numbers are given. Relatedness between (a) partial sequences (1181 bp) of the 16S rRNA gene and (b) partial sequences (599 bp) of the *tuf* gene. GenBank accession numbers for 16S rRNA gene sequences: LSPQ-04155 (KP793151), LSPQ-04156 (KP793152), LSPQ-04157 (KP793153) and LSPQ-04217 (KP793154). GenBank accession numbers for *tuf* gene sequences: LSPQ-04155 (KP793155), LSPQ-04156 (KP793156), LSPQ-04157 (KP793158) and LSPQ-04217 (KP793157). Bar, estimated number of substitutions per site.

**Fig. 2:**
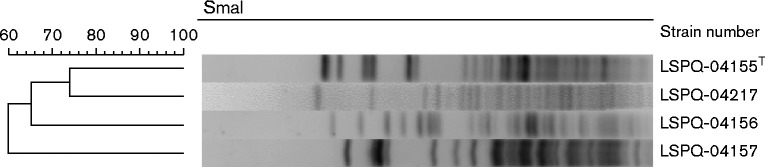
*Sma*I PGFE patterns of three *E. pallens* isolates compared with the reference strain LSPQ-04155 (ATCC BAA-351), identified with the letter ‘T’. The scale represents the percentage similarity between the isolates.

The three isolates were sent to the Laboratoire de santé publique du Québec (LSPQ) for identification. Classical biochemical testing, as well as 16S rRNA (Bekal *et al.*, 2006) and elongation factor (*tuf*) gene sequencing were performed. For *tuf* gene analysis, new primers were designed in order to amplify a 619 bp fragment of the gene from all the species of the genus *Enterococcus* (forward primer EntA1 5′-ATCTTAGTAGTTTCTGCTGCTGA, reverse primer EntA2 5′-GTAGAATTCAGGACGGTAGTTAG). The same primers were used for sequencing. Fig. 1 shows the phylogenetic trees of 16S rRNA and *tuf* gene sequences reconstructed with mega5 software (Tamura *et al.*, 2011). Multiple sequence alignment using the neighbour-joining method showed that isolates LSPQ-04155 (i.e. the type strain ATCC BAA-351), LSPQ-04156, LSPQ-04157 and LSPQ-04217 were closely related to each other, displaying 100 and 98 % similarity for 16S rRNA and *tuf*, respectively. Interestingly, the biochemical characteristics of the three isolates were identical to those of the type strain, with the exception of methyl-α-glucopyranoside which was positive, whilst the type strain was negative. The three isolates could not be identified using VITEK MS MALDI-TOF. The species *E. pallens* was not included in the VITEK MS IVD version 2.0 (bioMérieux), a clinically relevant species database, and SARAMIS version 4.1, the Research Use Only database. New spectra entries were added to the SARAMIS database using cultures from different times of incubation for each of the three isolates and also from the reference strain LSPQ-04155 (ATCC BAA-351). Based on this newly updated library, the SARAMIS database was able to identify the three isolates correctly to species level. The Bruker Biotyper database (Bruker Daltonics) contains the species *E. pallens*. The three strains were correctly identified with the Bruker Biotyper system. PFGE (Gagnon *et al.*, 2011) was performed and showed that all isolates had different PFGE patterns (Fig. 2). Susceptibility profiles to seven antimicrobials were obtained by broth microdilution according to Clinical and Laboratory Standards Institute guidelines (CLSI, 2014). All isolates were susceptible to penicillin, ampicillin, vancomycin, teicoplanin, quinupristin/dalfopristin, daptomycin and linezolid.

## Discussion

SBP is a common infection that affects patients with liver cirrhosis and ascites. The infection occurs following the translocation of gut microflora into the ascites and subsequent proliferation due to impaired host defence (Bennett *et al.*, 2015). Unsurprisingly, *Enterobacteriaceae*, in particular *E. coli* and *Klebsiella pneumoniae*, account for the majority of SBP. *Enterococcus* species, *Streptococcus pneumoniae* and other *Streptococcus* spp. are also common causative agents (Bennett *et al.*, 2015). The microbiology of SBP may be evolving in some regions. Reports of increasing prevalence of Gram-positive SBP in Turkey (Yakar *et al.*, 2010) and of the emergence of *Enterococcus* species SBP in Germany and in Greece have been published (Alexopoulou *et al.*, 2013; Reuken *et al.*, 2012). Here, we report the first series of patients who developed SBP due to *E. pallens*. In humans, this micro-organism had previously been isolated only once from the peritoneal dialysate of a patient with secondary peritonitis in Quebec (Tyrrell *et al.*, 2002). The fact that all four human infections described so far were peritonitis is noteworthy. Hence, *E. pallens*, as well as other *Enterococcus* species, appears to possess particular but as-yet unidentified virulence factors that favour the development of peritoneal fluid infections. Whether *E. pallens* preferentially colonizes the digestive tract of patients with cirrhosis or whether it colonizes humans regardless of underlying conditions but mainly causes infections in patients with cirrhosis remains to be determined. Also, the fact that all four *E. pallens* human infections reported to date in the literature originate from the province of Quebec raises the question of the geographical distribution of this micro-organism. However, as the four isolates analysed are not genetically related, the hypothesis of a single regional clone is unlikely. Further studies are needed to determine the prevalence of *E. pallens* and to better understand its virulence factors. Clinical microbiologist should be aware of this micro-organism which can be identified by phenotypic and molecular methods. As *E. pallens* and other *Enterococcus* species are intrinsically resistant to cephalosporins, clinicians should suspect these pathogens in the context of failure of first-line empirical agents.

In conclusion, we describe the first case series of human infections due to *E. pallens*. Together with the only previous reported human case, these suggest that *E. pallens* has a propensity to cause peritoneal fluid infections.
